# Evaluation of global terrestrial near‐surface wind speed simulated by CMIP6 models and their future projections

**DOI:** 10.1111/nyas.14910

**Published:** 2022-10-14

**Authors:** Cheng Shen, Jinlin Zha, Zhibo Li, Cesar Azorin‐Molina, Kaiqiang Deng, Lorenzo Minola, Deliang Chen

**Affiliations:** ^1^ Regional Climate Group, Department of Earth Sciences University of Gothenburg Gothenburg Sweden; ^2^ Key Laboratory of Atmospheric Environment and Processes in the Boundary Layer over the Low‐Latitude Plateau Region, Department of Atmospheric Science Yunnan University Kunming People's Republic of China; ^3^ Key Laboratory of Regional Climate and Environment for Temperate East Asia, Institute of Atmospheric Physics Chinese Academy of Sciences Beijing People's Republic of China; ^4^ Laboratory for Climate and Atmosphere‐Ocean Studies, Department of Atmospheric and Oceanic Sciences, School of Physics Peking University Beijing People's Republic of China; ^5^ Centro de Investigaciones sobre Desertificación, Consejo Superior de Investigaciones Científicas (CIDE, CSIC‐UV‐*Generalitat Valenciana*) Climate, Atmosphere and Ocean Laboratory (Climatoc‐Lab) Moncada Spain; ^6^ School of Atmospheric Sciences Sun Yat‐sen University, and Southern Marine Science and Engineering Guangdong Laboratory (Zhuhai) Zhuhai People's Republic of China; ^7^ Interuniversity Department of Regional and Urban Studies and Planning (DIST) Politecnico and University of Turin Turin Italy

**Keywords:** CMIP6, large ensembles, near‐surface wind speed, projections, windy days

## Abstract

We evaluate the performance of Coupled Model Intercomparison Project Phase 6 (CMIP6) models in simulating the observed global terrestrial near‐surface wind speed (NSWS) and project its future changes under three different Shared Socioeconomic Pathways (SSPs). Results show that the CESM2 has the best ability in reproducing the observed NSWS trends, although all models examined are generally not doing well. Based on projections of CESM2, the global NSWS will decrease from 2021 to 2100 under all three SSPs. The projected NSWS declines significantly over the north of 20°N, especially across North America, Europe, and the mid‐to‐high latitudes of Asia; meanwhile, it increases over the south of 20°N. Under SSP585, there would be more light‐windy days and fewer strong‐windy days than those under SSP245, which leads to a significant global NSWS decline. Robust hemispheric‐asymmetric changes in the NSWS could be due to the temperature gradient in the two hemispheres under global warming, with −1.2%, −3.5%, and −4.1% in the Northern Hemisphere, and 0.8%, 1.0%, and 1.5% in the Southern Hemisphere, for the near‐term (2021–2040), mid‐term (2041–2060), and long‐term (2081–2100), respectively.

## Graphical abstract

We evaluate the performance of Coupled Model Intercomparison Project Phase 6 (CMIP6) models in simulating the observed global terrestrial near‐surface wind speed (NSWS) and project its future changes under three different Shared Socioeconomic Pathways (SSPs) scenarios. Results show that the CESM2 has the best ability in reproducing the observed NSWS trends, although all models examined are generally not doing well.

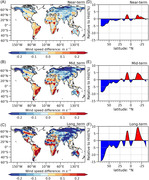

## INTRODUCTION

Near‐surface wind speed (NSWS) is a key parameter when studying atmospheric dynamics and climate change.^1^ Investigating how NSWS will change in the future is crucial for the evaluation and development of wind energy.^2^, ^3^ Moreover, changes in NSWS have a large impact on the environment and society by influencing extreme weather,^4^ atmospheric visibility,^5^ air quality,^6^ evapotranspiration,^1^ infrastructure,^7^ and crop production,^8^ among many others.

Roderick et al.^1^ discovered that terrestrial NSWS decreased in the past decades; such general slowdown was termed “stilling.” Vautard et al.^9^ investigated the NSWS changes at 822 stations across the globe and quantified that NSWS over the Northern Hemisphere (NH) mid‐latitudes decreased by 5–15% from 1979 to 2008. Besides, numerous studies have found that the declining trends in NSWS can be found at regional scales (e.g., Refs. 10, 11, 12, 13, 14). However, a reversal of the stilling occurred around 2010 in several regions of the world.^3^ The magnitudes and turning points of the stilling‐reversal phenomena display discrepancies among studies. Some recent studies proposed that the stilling and reversal could be a cyclical, decadal pattern of NSWS.^15^

For future projection, the Coupled Model Intercomparison Project (CMIP) models are widely used for regional‐scale studies. However, investigations about NSWS projections at a global scale are much fewer. Kumar et al.^16^ used the CMIP Phase 5 (CMIP5) to explore future extreme wind projections: they found a global decline in maximum wind speed by the end of the 21st century. Based on CMIP5 models, Karnauskas et al.^17^ revealed that the wind power (which is calculated by the NSWS) would decrease over the mid‐latitudes across the NH, while it would increase over the tropics and Southern Hemisphere (SH). Wu et al.^18^ found that CMIP Phase 6 (CMIP6) has a better ability in reproducing historical NSWS changes over China than CMIP5. Using the state‐of‐art CMIP6 models, Deng et al.^19^ studied the historical global NSWS changes and proposed that the Hadley circulation changes and interdecadal variability (Pacific Decadal Oscillation) could strongly affect the decadal NSWS changes. A recent study found that the large‐scale meridional temperature gradient has a significant effect on the hemispheric NSWS changes, such as enhanced high‐latitude warming over the NH could reduce the equator‐to‐pole surface air temperature (SAT) gradient.^20^ Wind blows are driven by a pressure gradient that, to a large extent, can be attributed to the uneven warming of the Earth's surface.^3^ Anthropogenic warming causes a spatially uneven increase in air temperature, which induces differences in air temperature gradient and subsequently changes in wind.^21^ Although Karnauskas et al.^17^ found that the projected global NSWS shows interhemispheric asymmetry based on 10 CMIP5 models, they did not estimate the performance of models in simulating the observed NSWS trends at the global and regional scales before making a projection based on CMIP5 models. Meanwhile, the uncertainties of the future NSWS changes based on different models are also not discussed. Although global climate models (GCMs) can reproduce the historical weakening characteristics of NSWS, most models significantly underestimate the long‐term weakening trend of NSWS.^22^ One way to reduce the projection uncertainty is to use those models that reasonably reproduce past trends, like what has been done for soil moisture projection, which has a similar issue.^23^ Because of that, evaluating the ability of all the models to reproduce past NSWS changes is an essential step before using GCMs for projection.

For all these reasons, this study aims at filling the knowledge gaps by (1) evaluating the performance of CMIP6‐coupled GCMs in simulating the historical changes of global and regional terrestrial NSWS, with a focus on the sign and magnitude of the past NSWS trend; (2) projecting the changes of global terrestrial NSWS under different greenhouse gas (GHG) emission scenarios based on CMIP6 GCMs with reasonable performance; and (3) quantifying uncertainties of the projections. The rest of this paper is organized as follows: datasets and methods are introduced in the second section and results are shown in the third section. The discussion is presented in the fourth section and conclusions are summarized in the fifth section.

## MATERIALS AND METHODS

### Datasets

#### Global Surface Summary of the Day database

To assess the performance of the CMIP6 models in reproducing the recent global terrestrial NSWS changes, we compare the simulated historical NSWS with the Global Surface Summary of the Day database. Based on data exchanged through the World Meteorological Organization World Weather Watch Program, the database is derived from the United States Air Force DATSAV3 surface dataset and the Federal Climate Complex Integrated Surface Hourly dataset.^3^ After the Air Weather Service runs the data through extensive automated quality control algorithms that correctly decode as much of the synoptic data as possible while eliminating most of the random data errors, these synoptic hourly observations are converted from recorded hourly data into mean daily values by the National Climatic Data Center.^24^, ^25^ To obtain a high‐quality NSWS record, Zeng et al.^3^ employed selection criteria to avoid including incomplete data series; with these metrics, they concluded that NSWS data recorded at 1435 stations provided the most useful information. In this study, we employ the data from those same 1435 stations (Figure S1).

#### CMIP6 datasets

CMIP6 multi‐model historical simulations (1850–2014) and projections (2015–2100) of monthly NSWS and SAT are used in this study. We also use daily NSWS from the best performance model to project windy days. This dataset includes multiple runs from various GCMs that are selected based on their CMIP6 “ripf” indices. Each ripf index represents the initial state (realization r), the initialization method (i), the physics version (p), and the forcing dataset (f).^26^ Note that only the first ensemble member (index of “r1i1p1f1”) is selected from each model.^27^

The names and horizontal resolutions of 22 CMIP6 models are listed in Table S1. The spatial resolutions and physical parameterizations in CMIP6 have been improved compared to those in CMIP5.^28^ The model performance is usually evaluated by comparing the simulation with the observations; we selected 1978–2014 as the historical period because models and observations share the same period in the past. For NSWS projections, 22 models are analyzed with Shared Socioeconomic Pathways (SSPs) scenarios 245, 370, and 585;^29^ these scenarios result in an end‐of‐century radiative forcing of 4.5, 7.0, and 8.5 W m^−2^, respectively.^30^ The SSPs represent a range of future GHG emissions and land‐use and land‐cover change (LUCC) scenarios estimated from integrated assessment models and based on various assumptions regarding economic growth, climate mitigation efforts, and global governance.^29^

#### Large ensemble dataset

The state‐of‐art large ensembles (LEs) dataset from the Max Planck Institute Earth System Model (MPI‐ESM) is used to evaluate the effect of external forcing on the detected NSWS changes and the consistency of the results. The LEs dataset of MPI‐ESM includes 100‐member ensembles of simulations and has been evaluated by numerous studies (e.g., Refs 20, 31, and 32). The MPI‐ESM is a fully coupled atmosphere–ocean model, including land and ocean biogeochemistry processes, with a horizontal resolution of T63 (approximately 1.9° × 1.9°) and 47 vertical layers up to 0.01 hPa.^32^ Individual members of the MPI‐ESM only differ in their initial conditions.^33^ The historical simulations are integrated from 1850 to 2005, driven by observed radiative forcing. The future simulations from 2006 to 2099 are used in this study. Given that the strong warming magnitude could not be reached with the low emission scenario, the radiative forcing from the representative concentration pathway 8.5 (RCP8.5) scenario in MPI‐ESM LEs is used. The MPI‐ESM LEs have been extensively used to understand the effects of internal climate variability and external forcing on climate projections.^34^, ^35^

## METHODS

### Piecewise linear function

The traditional single linear model cannot provide an adequate description of the change in tendency during different periods, so we employ a piecewise linear function (PWLF) to statistically analyze the NSWS trends. A PWLF automatically detects where the slope of a linear function changes, identifies the optimal breakpoints, and allows multiple linear models to be fitted to each distinct section of the time series.^36^ A PWLF is expressed as Equation (1):

(1) $$y\left(t\right)\;=\left\{\begin{array}{c} \alpha_{1}+\beta_{1}\left(t-b_{1}\right)\;\;b_{1}<t<b_{2}\\ \alpha_{2}+\beta_{2}\left(t-b_{2}\right)\;\;b_{2}<t<b_{3}\\ \vdots \\ \alpha_{n_{b}-1}+\beta_{n_{b}-1}\left(t-b_{n_{b}-1}\right)\;b_{n_{b}-1}<t<b_{n_{b}} \end{array},\right.\;\;$$

where *b*_1_ is the location of the first breakpoint, *b*_2_ is the location of the second breakpoint, and so forth until the last breakpoint $b_{n_{b}}$. In cases when the breakpoint locations are unknown, optimization is used to find the best set of breakpoints that minimize the overall sum of the square of the residuals. In the PWLF, we utilize the differential evolution algorithm for global optimization.^37^ This algorithm is a popular heuristic optimizer that has been used in a wide variety of applications.^36^

### Estimating model performance indices

The significance of future changes predicted from climate models can be measured by the model's credibility in simulating present‐day climate.^34^ The most common method of assessing a climate model is the quantitative assessment of how well the simulated results reproduce the observational data.^38^ The performance of the CMIP6 models in simulating the observed NSWS is assessed by the wind speed bias (Bias; $Bias\;=y^{m}\;-y^{o}$, $\mathrm{where}\;y^{m}$ and $y^{o}$ denote the CMIP6 and observation, respectively). The linear trend is calculated based on the least square method.

### SAT difference between high and low latitudes

Following Zha et al.,^20^ four latitude zones are defined as: area1 (60°N–90°N, 180°W–180°E), area2 (0–60°N, 180°W–180°E), area3 (45°S–90°S, 180°W–180°E), and area4 (0–45°S, 180°W–180°E). The SAT difference (SATD) is then calculated as follows:

(2) $$\;SATD_{NH}=SAT_{area1}\;-\;SAT_{area2}\;$$

(3) $$\;SATD_{SH}=SAT_{area3}\;-\;SAT_{area4}.$$

### Other methods

To quantify the difference in NSWS between future and present time, we assess the relative changes in the future NSWS with respect to observation in the present using Equation (4):

(4) $$W\;=\frac{U_{f}-U_{p}}{U_{p}}\;\times 100\%,$$

where $U_{f}$and $U_{p}$ denote the future NSWS and present NSWS, respectively. We focus on four specific periods: 1995–2014 (the “present”), 2021–2040 (the “near‐term”), 2041–2060 (the “mid‐term”), and 2081–2100 (the “long‐term”). Prior to our analysis, we regrid the CMIP6 NSWS and SAT datasets onto a common 1° × 1° regular grid resolution using a bilinear interpolation technique and an ocean mask.^17^, ^39^ To evaluate future changes in the occurrence of different categories of windy days, four windy‐day thresholds are defined in terms of their daily wind speed percentiles in the “present” period based on model simulation: light windy days (<25th percentile), gentle windy days (between 25th and 50th percentiles), moderate windy days (between 50th and 75th percentiles), and strong windy days (>75th percentile).^40^ To estimate the regional differences of global NSWS, the globe is divided into different subregions:^41^, ^42^ North America, South America, Europe, Central Asia, South Asia, East Asia, Australia, and Africa (see Figure S1 for details).

## RESULTS

### Evaluation of CMIP6 performance in simulating the observed NSWS

**FIGURE 1:**
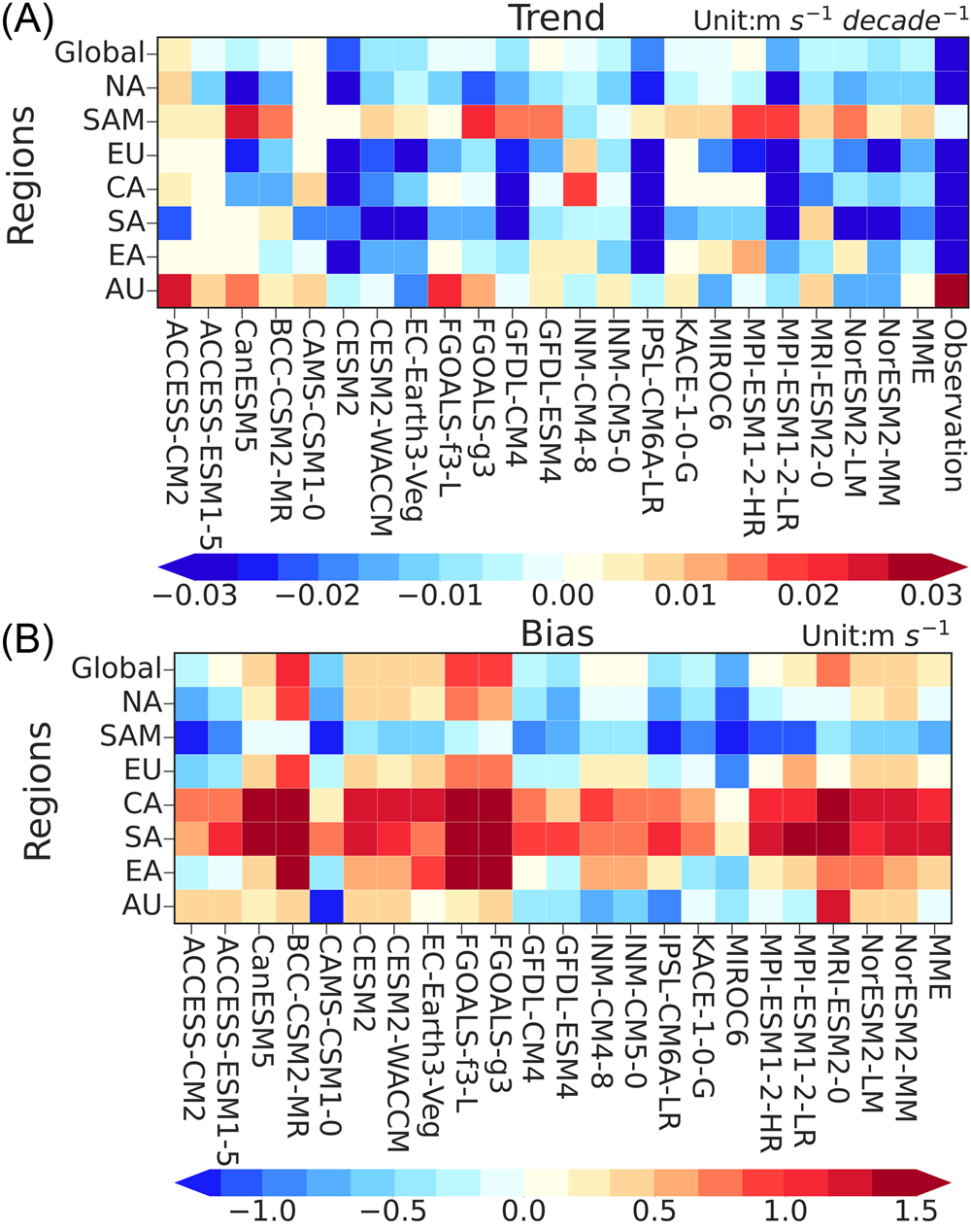
Heat maps of (A) the wind speed trend in 22 CMIP6 models and the multi‐model ensemble (MME) and (B) the wind speed bias between simulated and observed NSWS from 1978 to 2014. Abbreviations: AU, Australia; CA, Central Asia; EA, East Asia; EU, Europe; NA, North America; SAM, South America; SA, South Asia.

We assess the performance of 22 CMIP6 models with respect to their abilities in reproducing the historical global terrestrial NSWS changes during 1978–2014. Most of the 22 CMIP6 models underestimate the magnitudes of the NSWS over North America and South America and overestimate the NSWS over Central Asia, South Asia, and East Asia (Figure 1B). Most CMIP6 models are capable of capturing the observed NSWS decreases. However, the magnitude of the reduction in NSWS is underestimated, which will be further discussed in the following section. In all CMIP6 models, the largest NSWS decline is found in the CESM2 (−0.021 m s^−1^ decade^−1^; *p* < 0.10) (Figure 1A). We focused on the model's ability in reproducing the NSWS trends, specifically the stilling and the reversal phenomena. As a result, the CESM2 outperforms the other models when it comes to simulating the past global terrestrial NSWS trends as well as their reversals.

**FIGURE 2:**
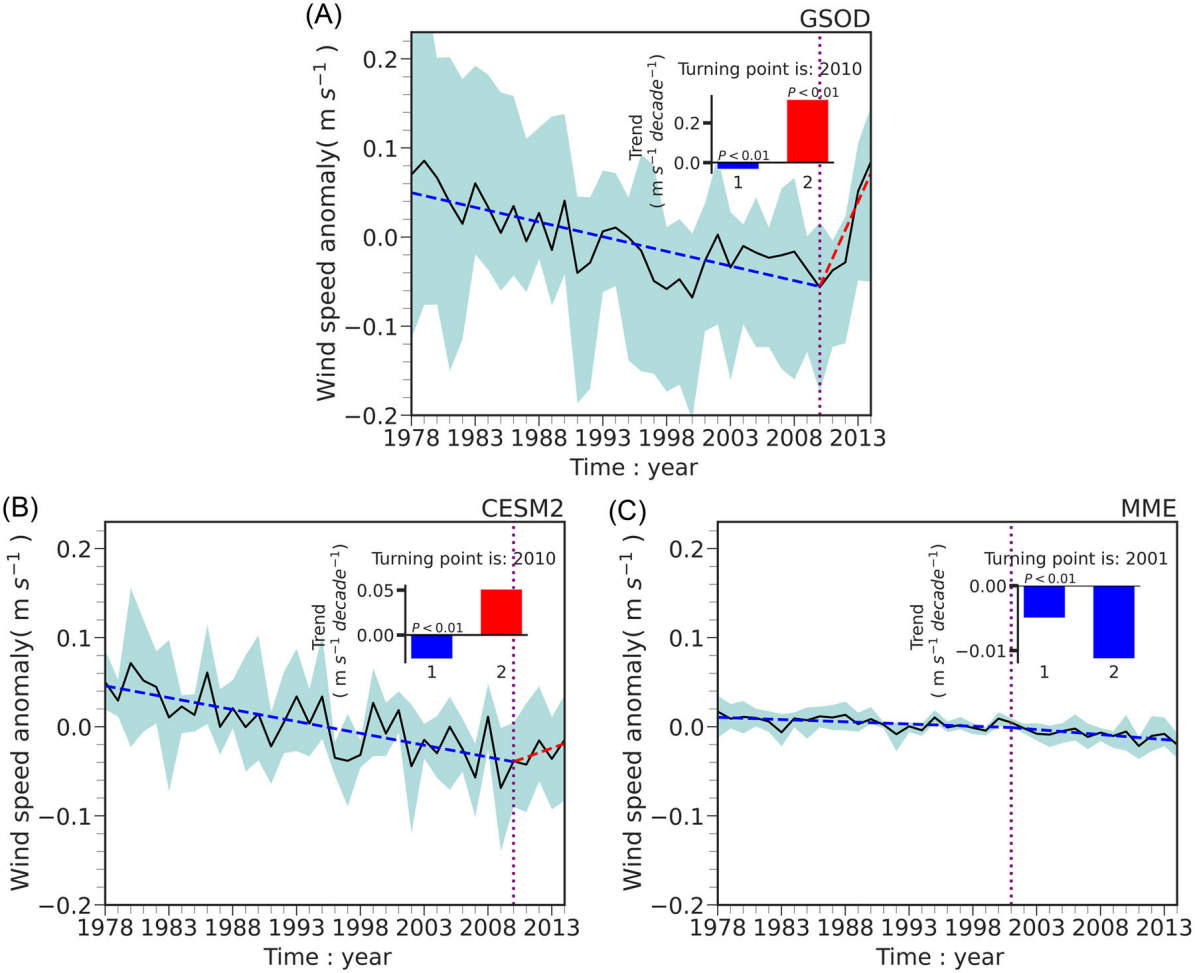
Temporal evolutions of the NSWS anomaly in (A) the observation, (B) the CESM2, and (C) the multi‐model ensemble (MME) of 22 CMIP6 models. The shaded area indicates the likely range (25th–75th percentiles). The blue and red dotted lines represent the piecewise linear function (PWLF) fittings. The trends for these two periods are shown in the inset. *p* < 0.01 indicates that the goodness‐of‐fit of the PWLF passes the *t*‐test significance at the 0.01 level.

**FIGURE 3:**
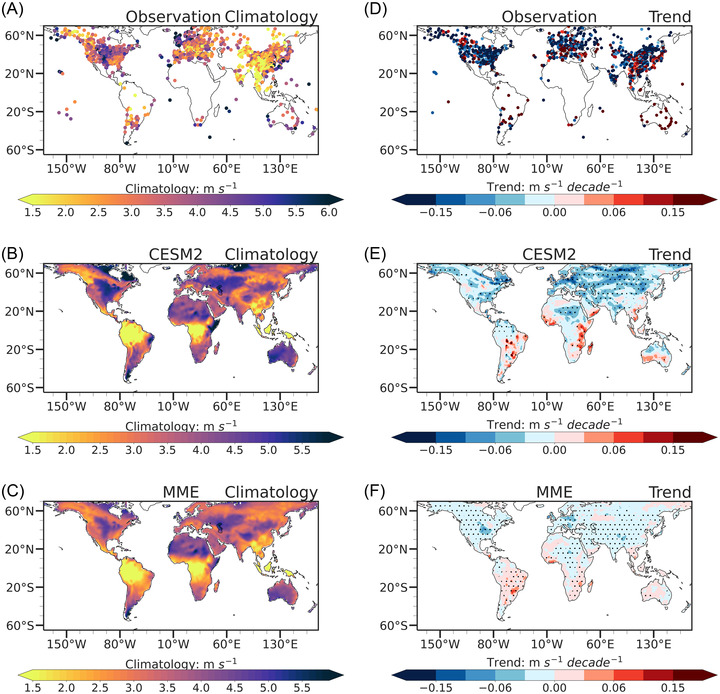
Spatial patterns of the mean NSWS (A–C) and the NSWS trends (D–F) from 1978 to 2014. (A) and (D) for observation, (B) and (E) for CESM2 model, and (C) and (F) for the multi‐model ensemble (MME) of the 22 CMIP6 models. Dots in (D), (E), and (F) denote the trends that pass the *t‐*test significance at the 0.10 level.

The temporal evolutions of NSWS in the observations, CESM2, and the multi‐model ensemble (MME) of the CMIP6 models for the historical period (1978–2014) are also compared (Figure 2). The observational data indicate that the NSWS decreased during 1978–2010 and increased during 2011–2014. The statistical fitting of the PWLF during two periods passes the *t‐*test significance at the 0.01 level (Figure 2A). The stilling and reversal are also reproduced by CESM2 (Figure 2B), although the magnitudes of trends are weaker than those found in observations. This stilling‐reversal phenomenon is not captured by the MME (Figure 2C). The fluctuations in the interquartile ranges of NSWS are also more pronounced in the CESM2 than those in MME. The spatial patterns of climatology and trends in the observation, CESM2, and MME of CMIP6 are also evaluated (Figure 3). Results show that the means in NSWS in CESM2 and MME are consistent with the observations (Figure 3A–C). The observed NSWS reduction from 1978 to 2014 has been reproduced by CESM2, specifically in North America, Europe, and Asia, where the downward trends pass the *t‐*test significance at the 0.10 level (Figure 3E). The MME of CMIP6 also captures the decline in NSWS over the mid‐latitudes of the NH, but the magnitude of the decline in the MME of CMIP6 is less than that in the CESM2 (Figure 3F). As CESM2 has reasonably reproduced the stilling and the reversal phenomena, this study will evaluate the future changes in global terrestrial NSWS primarily based on the CESM2 projections.

### Projected future changes in global terrestrial NSWS with different SSPs

**FIGURE 4:**
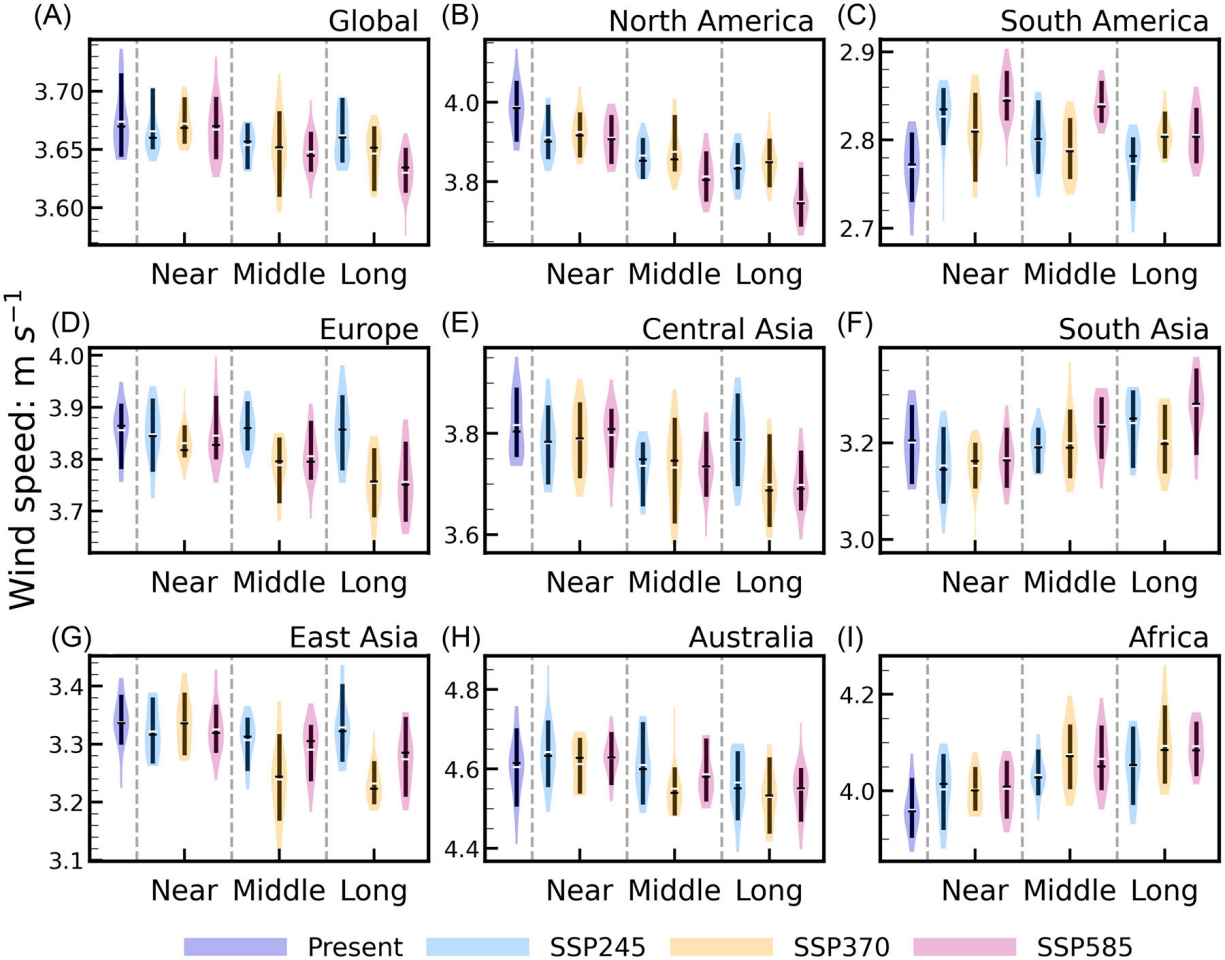
(A) Global and (B–I) regional violin plots of the projected NSWS for SSP245, SSP370, and SSP585 in the near‐term, mid‐term, and long‐term. Present period is 1995–2014. Black lines represent the mean value, white lines represent the median value, and the whiskers correspond to the 10th and 90th percentiles of the NSWS. The shaded area represents the entire distribution of the data. The wider sections of the violin plot correspond to an increased likelihood that a given NSWS value will occur, while the thinner sections correspond to a lower likelihood of achieving a given NSWS.

We mainly focus on the decadal changes of the NSWS in this study. In particular, we use a violin plot to illustrate the distributions and changes in the future NSWS (Figure 4). Globally, the NSWSs do not appear to be affected considerably by the different levels of emissions in the near‐term (2021–2040) and the mid‐term (2041–2060). However, for the long‐term (2081–2100), the increase in emissions may force a larger decrease in NSWS (Figure 4A). Besides, it is worth noting that the projected changes in NSWS are not significantly correlated with the emissions in several regions, such as South America, Europe, Central Asia, East Asia, and Australia, implying that the physical mechanisms that drive NSWS changes could be different across the world under global warming.

**FIGURE 5:**
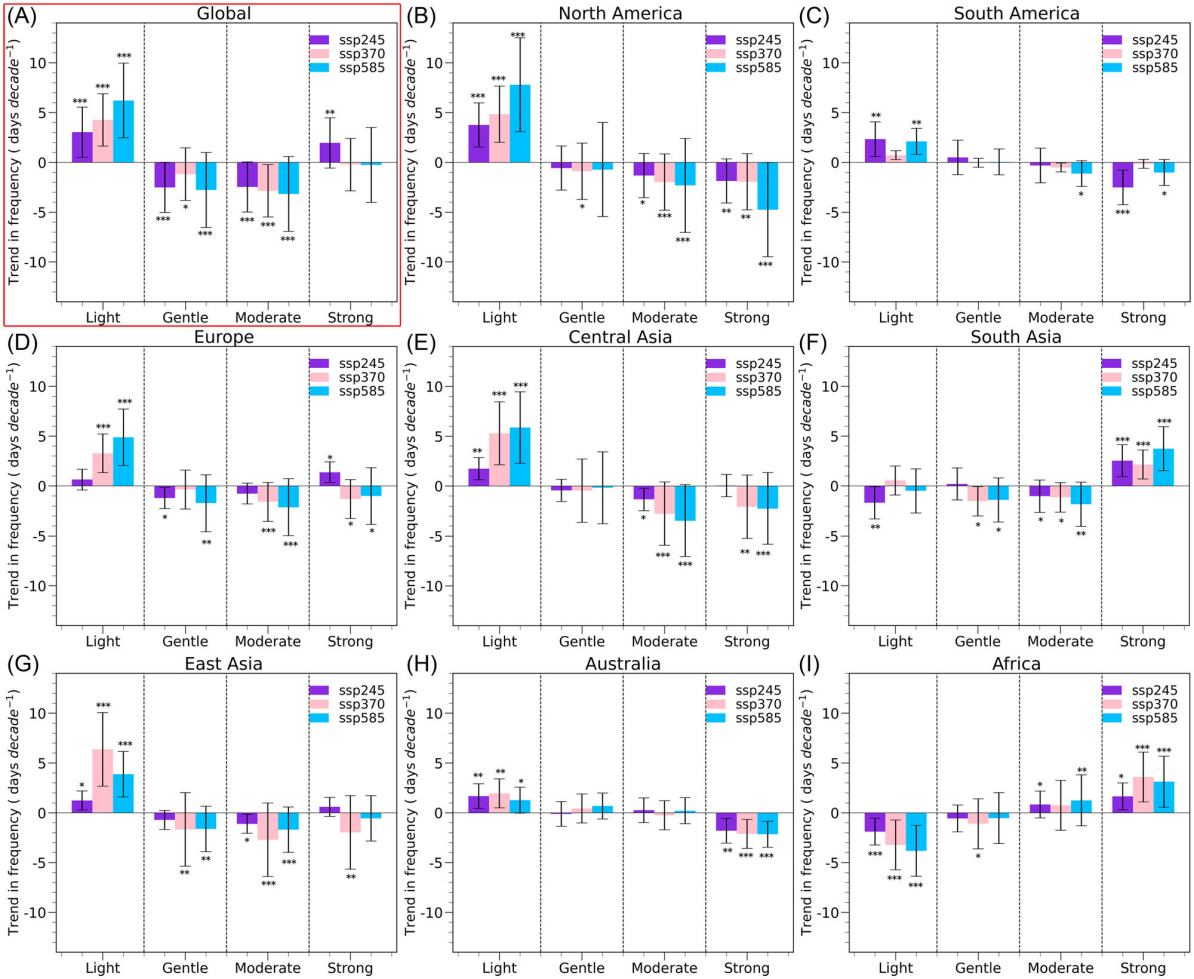
(A) Global and (B–I) regional linear trends in the number of light, gentle, moderate, and strong windy days for SSP245, SSP370, and SSP585 from 2021 to 2100. *, **, and *** denote the trends that pass the *t‐*test significance at the 0.10, 0.05, and 0.01 levels, respectively.

To determine whether decadal NSWS changes are affected by different types of windy days, trends in the frequency of light, gentle, moderate, and strong windy days from 2021 to 2100 are analyzed (Figure 5). Globally, the numbers of light windy days increase under the forcing scenarios of SSP245 (+3.0 days decade^−1^; *p* < 0.001), SSP370 (+4.3 days decade^−1^; *p* < 0.001), and SSP585 (+6.2 days decade^−1^; *p* < 0.001). The frequency of gentle, moderate, and strong windy days decreases in all SSPs, except for the number of strong windy days in SSP245; meanwhile, the frequency of light windy days increases with the strengthening of emission (Figure 5A). Accordingly, the slowdown in the global terrestrial NSWS for 2021–2100 is largely caused by the reduction in the number of gentle and moderate windy days. There are regional differences in the number of different types of windy days. We attribute the decrease in the NSWS over North America (Figure 5B), South America (Figure 5C), and Australia (Figure 5H) to the decreasing number of strong windy days. But the weakened NSWS over Europe (Figure 5D), Central Asia (Figure 5E), and East Asia (Figure 5G) are due to the decreased number of all wind days except light windy days. The increase in NSWS over South Asia (Figure 5F) and Africa (Figure 5I) corresponds to a significant increase in the number of strong windy days. Selecting SSP585 over SSP245 would result in additional growth in the number of strong windy days in South Asia (+1.6 days decade^−1^) and Africa (+1.5 days decade^−1^). Therefore, the external forcing (GHG emission) enhancement affects the changes in windy events, but these two parameters are not linearly related to one another.

### Projected asymmetry changes in terrestrial NSWS between two hemispheres

**FIGURE 6:**
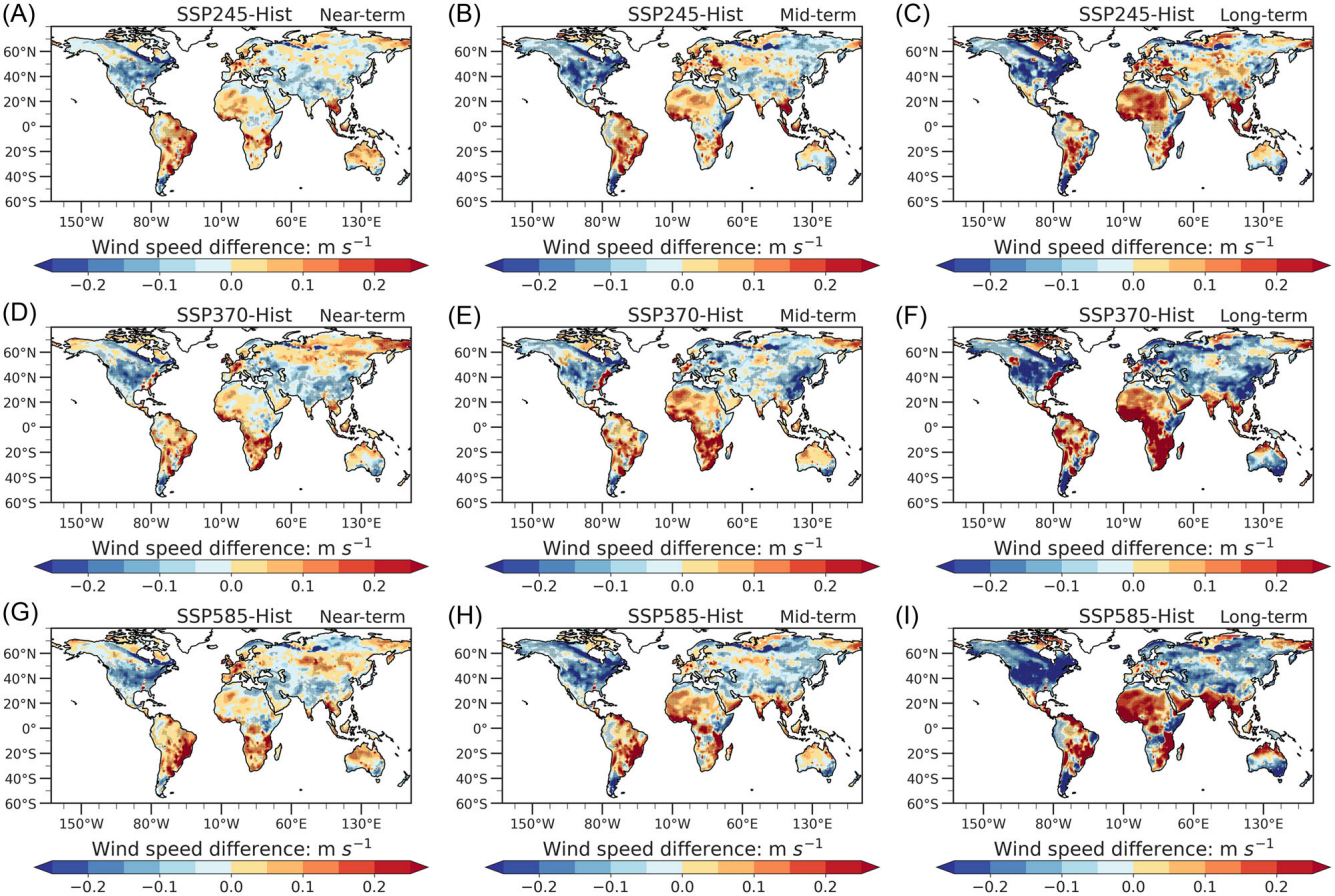
Spatial patterns of the difference between the future and present‐day NSWS during (A, D, G) near‐term, (B, E, H) mid‐term, and (C, F, I) long‐term periods under (A, B, C) SSP245, (D, E, F) SSP370, and (G, H, I) SSP585. The shaded area represents the results that pass the *t*‐test significance at the 0.10 level.

We quantify the differences in NSWS between the historical and the future period under different SSPs to investigate how the SSPs affect NSWS changes. With a “present‐day” baseline period (1995–2014), we calculate the NSWS difference between the future and the present day (denoted by NSWSD hereinafter) for three SSPs (Figure 6). We find negative NSWSD over North America, Europe, and Asia, and an increase in NSWSD over South America, Africa, and Southeast Asia. For SSP245 (Figure 6A–C), the reduction of NSWSD over North America is more significant during the long‐term than that during the near‐term, which is more than −0.2 m s^−1^ (*p* < 0.10). Furthermore, the increase in NSWSD over the south of 20°N is stronger in the long‐term than that in the near‐term and exceeds 0.2 m s^−1^ (*p* < 0.10) in most of South America and North Africa. The above characteristics in NSWS are more evident in SSP370 (Figure 6D–F) and SSP585 (Figure 6G–I). The NSWSD changes are more remarkable in SSP585 than those in SSP245. In conclusion, higher GHG emissions tend to enhance the asymmetry in the NSWS changes between the two hemispheres.

**FIGURE 7:**
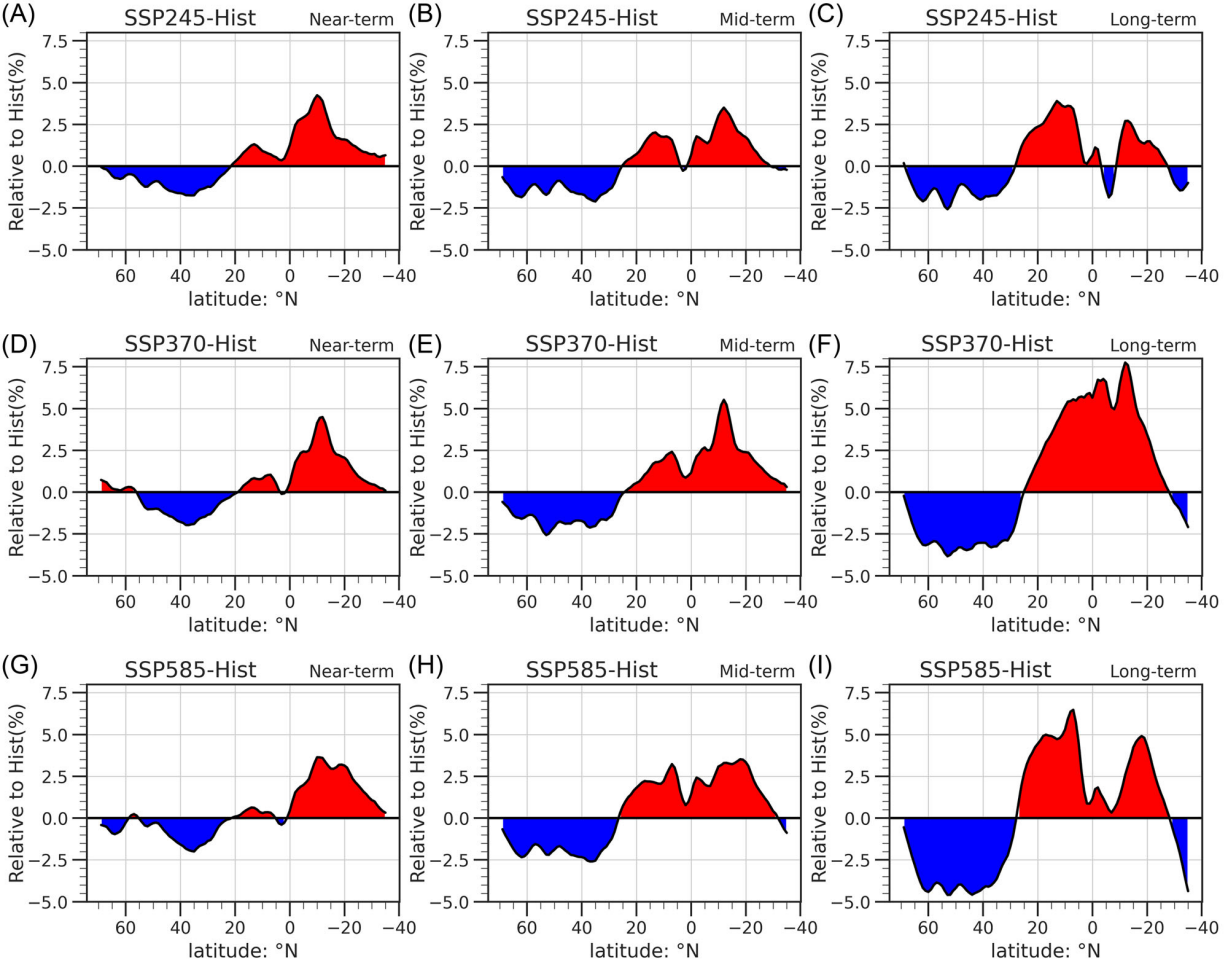
Relative to historical (1995–2014) changes of zonal mean NSWS during (A, D, G) near‐term, (B, E, H) mid‐term, and (C, F, I) long‐term periods under (A–C) SSP245, (D–F) SSP370, and (G– I) SSP585. Horizontal axis denotes the latitudes, and the vertical axis denotes the percentage changes (%) in NSWS in the future relative to historical NSWS.

We calculate the relative changes in future zonal‐mean NSWS compared to the present day during different periods (Figure 7) to quantify the magnitudes of changes. For all SSPs, there are negative values of relative changes over the north of 20°N and positive values of relative changes over the south of 20°N, with the larger magnitudes existing under higher emissions and for a longer term. The largest decrease is −2.5% in SSP245 (Figure 7A–C), but it reaches −4.9% in SSP585 (Figure 7G–I). For the area south of 20°N, the increase in SSP585 (>3.0%) is larger than the one in SSP245 (∼3.0%). While there is a little difference between the relative NSWS changes in the near‐term and the mid‐term for SSP245 and SSP585, the differences are larger for SSP585 than for SSP245 during the long‐term. It is worth noting that the projected NSWS changes in the SH under SSP370 are larger than those under SSP585. This may be due to dynamics that play a role in NSWS changes, which would lead to the possibility of nonlinear changes in projected changes.

### Factors contributing to asymmetric NSWS changes between two hemispheres

**FIGURE 8:**
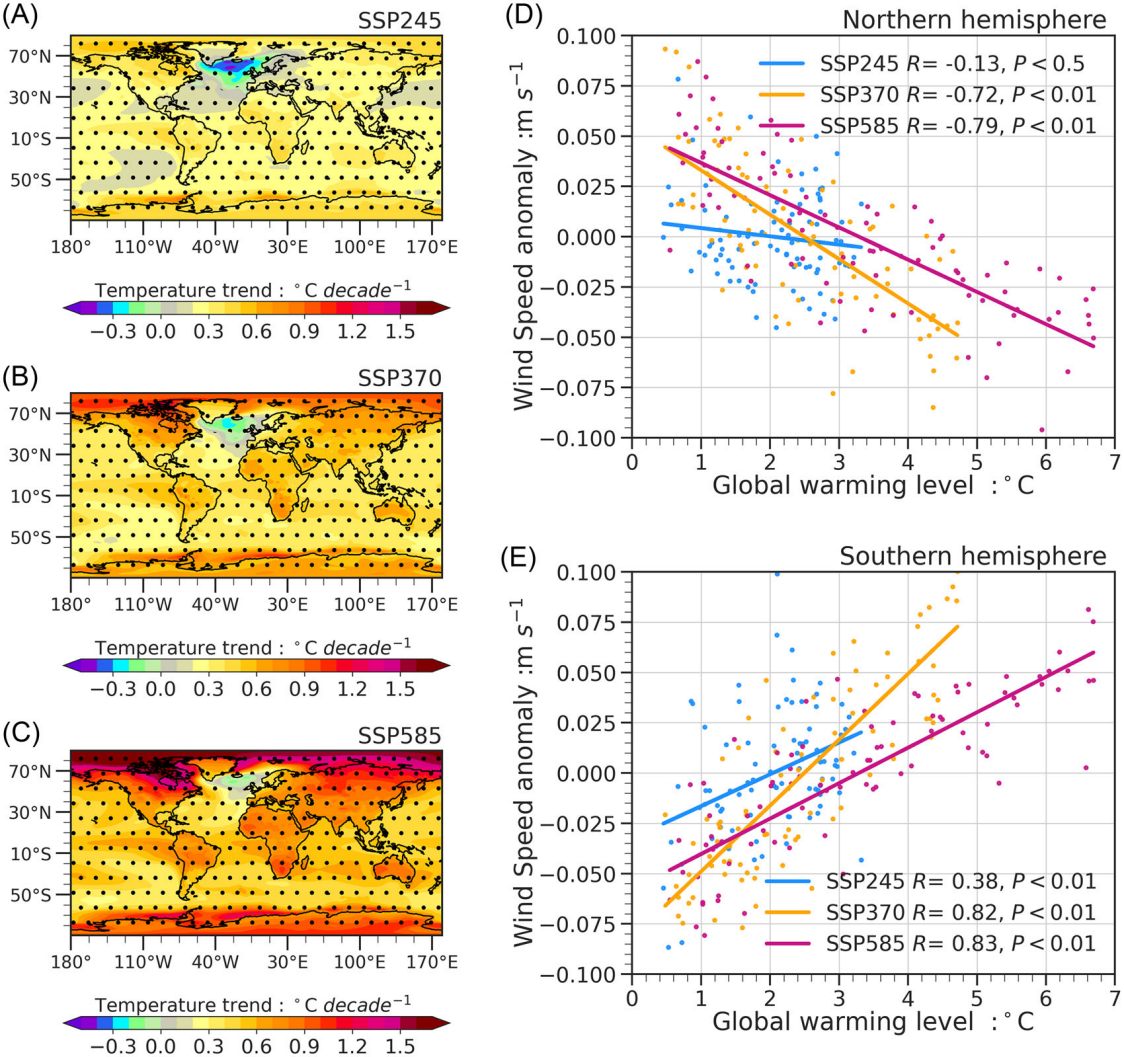
Spatial patterns of SAT trends in (A) SSP245, (B) SSP370, and (C) SSP585 from 2021 to 2100, and the scatter diagrams between SAT and NSWS anomaly (the difference between future annual mean and the climatology in 1995–2014, which represents the present‐day) over (D) the Northern Hemisphere and (E) Southern Hemisphere from 2021 to 2100. Dots in (A), (B), and (C) denote the trends that pass the *t‐*test significance at the 0.10 level. In (D) and (E), lines denote the linear fitting, *R* denotes the correlation coefficient, and *p* is the significance level.

**FIGURE 9:**
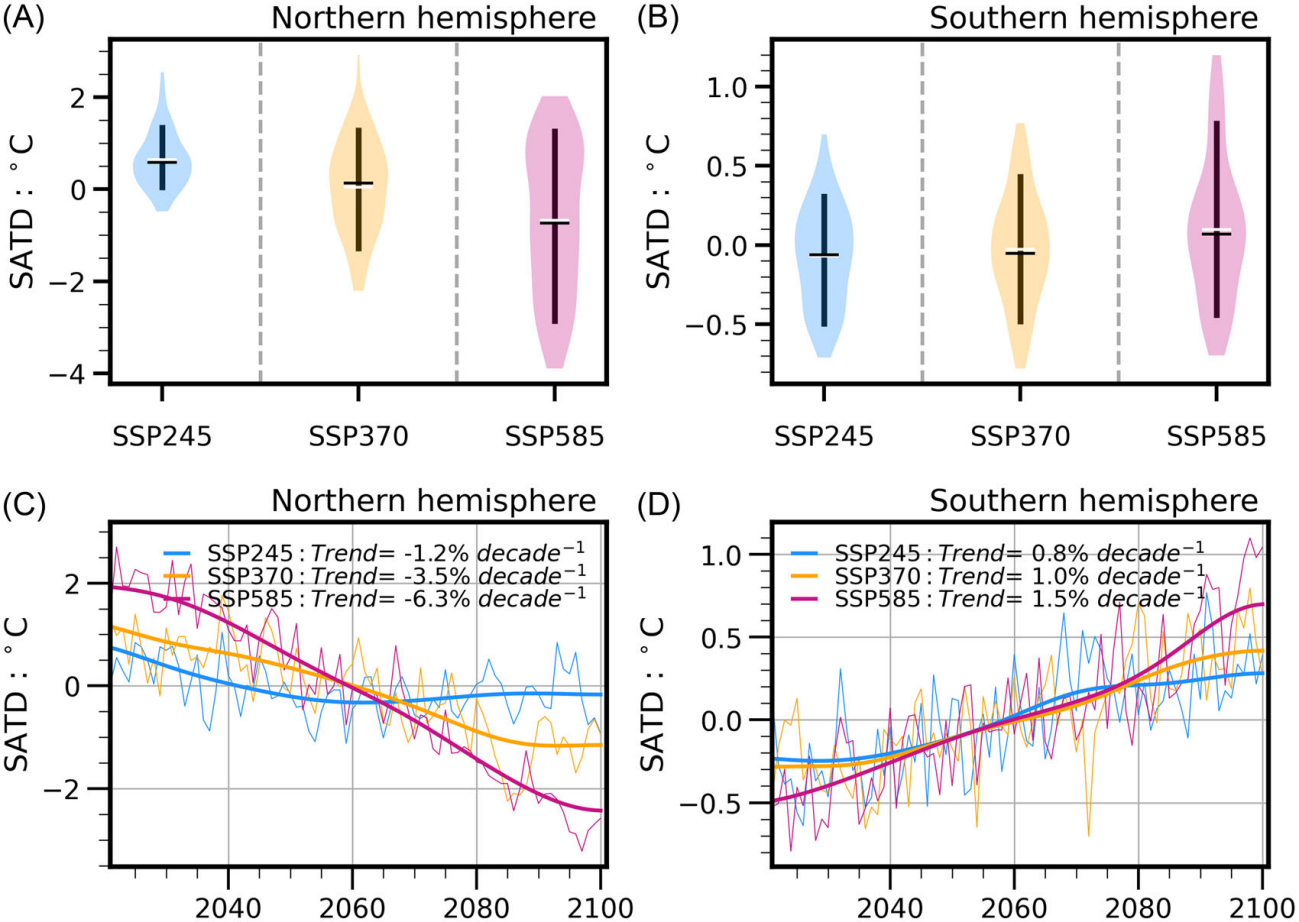
Violin plots of SAT difference between high and low latitudes (denoted by SATD) over the (A) Northern Hemisphere and (B) Southern Hemisphere, as well as temporal evolutions of SATD anomaly over the (C) Northern Hemisphere and (D) Southern Hemisphere in SSP245, SSP370, and SS585. In (A) and (B), black and white lines show the mean and median values, respectively. The whiskers show the 10th and 90th percentiles of SATD. Shaded area indicates the full distribution of the data. The wider sections of the violin plot represent a higher probability of SATD taking a given value, the thinner sections correspond to a lower probability. Trends in (C) and (D) denote the trends of SATD anomalies, which pass the *t‐*test significance at the 0.10 level.

Global SAT exhibits an increasing trend that passes the *t‐*test significance at the 0.10 level for all three SSPs, and the weakest and strongest SAT increases are generated by SSP245 (Figure 8A) and SSP585 (Figure 8C), respectively. For SSP370 and SSP585, the increase in SAT is largest over the high latitudes of the NH. We confirm that the NSWS changes and the global warming levels in the future show a negative correlation over the NH and a positive correlation over the SH. The correlation coefficients between NSWS and SAT are more significant in SSP370 and SSP585 than those in SSP245 (Figure 8D,E). Furthermore, we calculate the SATD between the low and high latitudes in these two hemispheres. Over the NH (SH), under SSP585, the mean SATD and the magnitude of the SATD 10th percentile are 1.45% (0.34%) and 3.28% (1.12%) lower than the values found using SSP245, respectively. As is shown, the change in the SAT gradient is more significant for the NH than it is for the SH due to stronger GHG forcing (Figure 9A,B). As for the temporal evolutions of SATD, the magnitude of the SAT gradient decreases for all SSPs (Figure 9C,D). The magnitudes of the SAT gradient will experience a significant decline with SSP370 and SSP585. The SSP585 results in a reduction of 6.3% in SAT gradient, while it is 1.2% for SSP245 over the NH. Besides, in SSP585, the magnitude of the decrease in SAT gradient is much larger over the NH than it is over the SH. In short, we conclude that the higher emissions could cause a more significant difference in SAT gradient between the two hemispheres, which induces a more significant decrease in NSWS over the NH and a weaker increase in NSWS over the SH. In a recent study, Deng et al.^43^ also found that the SH westerlies will be enhanced under global warming, which is induced by the changes in the equator‐to‐pole air temperature gradient. Besides, tropospheric circulation changes related to some natural internal variabilities (such as North Atlantic Oscillation and Interdecadal Pacific Oscillation, etc.) may have key contributions to local NSWS changes.^3^

### Comparisons of results in other good performance models

Based on CESM2, the abovementioned results show that the projected terrestrial NSWS would weaken over the mid‐to‐high latitudes of the NH and strengthen over the south of 20°N. The projected decline and increase in NSWS become more significant under a higher SSP. However, these results are obtained based on a single model. Different models are driven by different physical parameterization schemes and dynamic frameworks. The response intensities of different models to external forcing show discrepancies^44^ and the projection uncertainty of CESM2 must be discussed. To evaluate whether the projected asymmetry changes in terrestrial NSWS between two hemispheres can be reproduced in other models, we also compare the results in CESM2 with other models that show good performance in simulating the historical NSWS. Based on Figure 1, the CESM2‐WACCM and MIROC6 are also picked out. The results show that the projected asymmetry changes in terrestrial NSWS between two hemispheres based on CESM2 are also reproduced in CESM2‐WACCM (Figure S2) and MIROC6 (Figure S3), although spatial discrepancies are found among models. Compared to CESM2, the NSWS over the mid‐to‐high latitudes of the NH shows a stronger decrease in CESM2‐WACCM (Figure S4) and a weaker decrease in MIROC6 (Figure S5). Similarly, the NSWS over the south of 20°N also shows weaker and stronger increases in CESM2‐WACCM (Figure S4) and MIROC6 (Figure S5), respectively. The discrepancies in results could be mainly caused by internal variability^45^, ^46^, ^47^ and the model resolution.^42^ We note that the GCMs can reproduce the decreasing trend of historical NSWS, and the projected future NSWS shows a hemispheric‐asymmetry change.

### Elimination of internal variability in the effect of NSWS changes

Results from different single models show that the projected future changes in global NSWS are mainly attributed to intensified emission, which is considered an external forcing. However, uncertainty from model internal variability exists among the models. Some previous studies employ the MME of CMIP5/6 to eliminate the effects of internal variability and model bias.^16^, ^17^ The projected results show large uncertainty based on MME because the internal variability and other factors cannot be well excluded.^47^ Recent studies have pointed out that the effects of internal variability, model bias, and differences in initial condition can be eliminated based on the ensemble mean of LEs.^46^, ^48^ Therefore, the effects of external forcing (e.g., GHG‐induced global warming) on NSWS can be effectively extracted based on the member mean of LEs.

**FIGURE 10:**
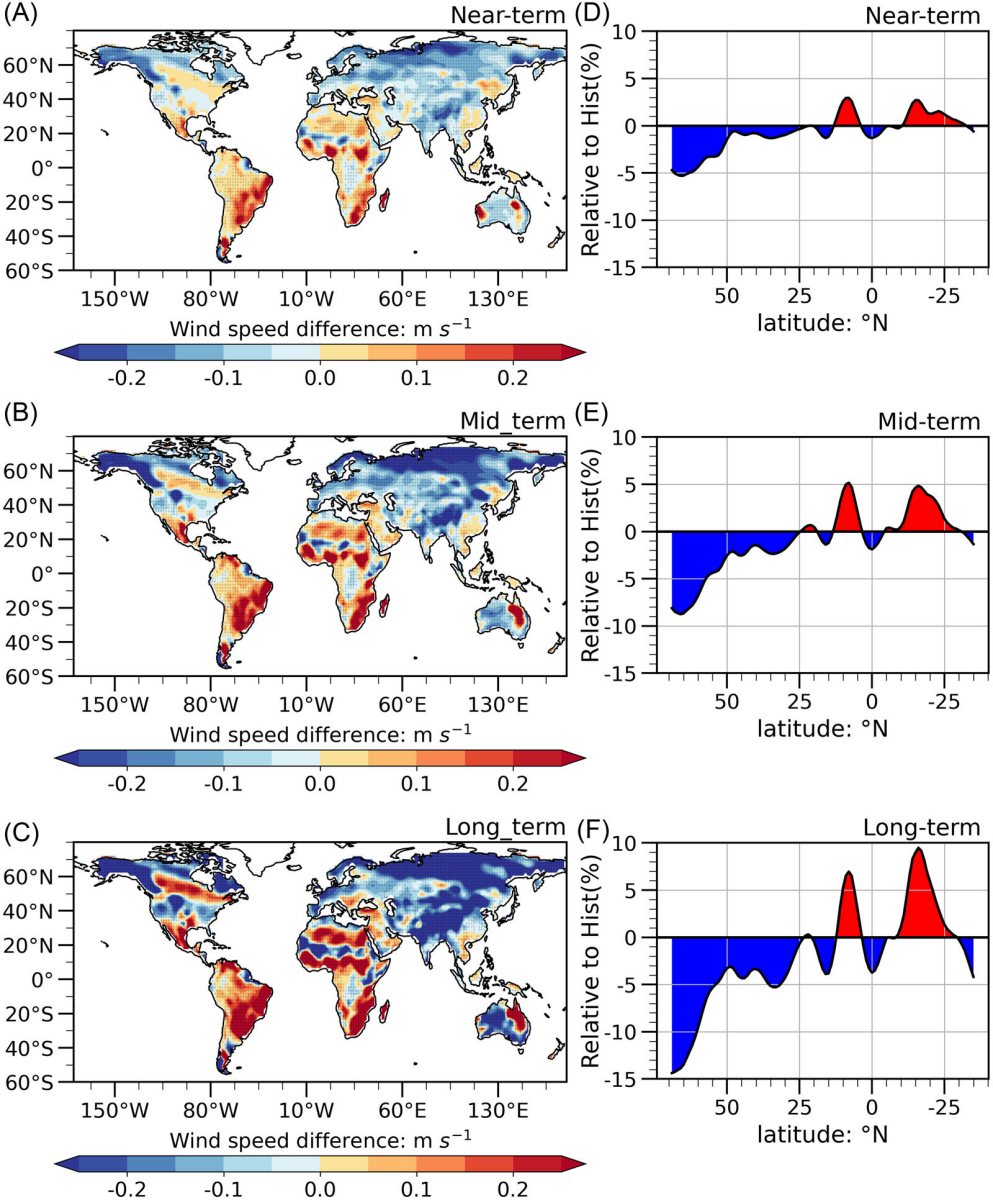
(A–C) Spatial patterns of the difference between the future and present‐day NSWS for RCP8.5 scenario based on ensemble mean of 100 members in MPI‐ESM, and (D–F) the relative to historical (1995–2014) change of zonal mean NSWS during (D) near‐term, (E) mid‐term, and (F) long‐term periods under RCP8.5 scenario based on ensemble mean of 100 members in MPI‐ESM. In (A), (B), and (C), the shaded area represents the results that pass the *t‐*test significance at the 0.10 level. In (D), (E), and (F), the horizontal axis denotes the latitudes, and the vertical axis denotes the relative historical changes in NSWS in the future.

Here, we employ the MPI‐ESM LEs to evaluate the effect of external forcing. The results show that the NSWS decreases over mid‐to‐high latitudes of the NH and increases over Eurasia, North America, and Africa (Figure 10A–C). Spatial patterns of the differences between the future and present‐day NSWSs based on MPI‐ESM LEs are similar to that shown in Figure 6. Furthermore, the relative changes in future NSWS compared to the present day at the different latitudinal bands are calculated (Figure 10D–F). The negative values over the north of 20°N and the positive values over the south of 20°N are also simulated. Moreover, the magnitudes of the relative change increase from the near‐term period to the long‐term period. Therefore, it is quite robust that the asymmetric changes in NSWS between two hemispheres could be enhanced by the increased emissions, and the effect from internal variability is small.

Based on the above results, we propose that the strengthening of GHG emissions could cause a decrease in NSWS over the mid‐to‐high latitudes of the NH and an increase in NSWS over the south of 20°N. This feature could be more pronounced in the long‐term than in the near‐term. Still, factors like internal variability, model bias, and differences in initial‐condition among models could affect the contribution of GHG emissions. For the strongest emission scenario (SSP585), these noises are responsible over the mid‐to‐high latitudes of the NH for an NSWS underestimation of 1.29%, 1.89%, and 3.03% for near‐term, mid‐term, and long‐term, respectively. Meanwhile, such underestimation increases over the south of 20°N, with values of 0.03%, 0.22%, and 1.63% for near‐term, mid‐term, and long‐term, respectively.

## DISCUSSION

### Potential reasons why CMIP6 models underestimate the reduction in NSWS

The observed decreasing trend in NSWS is underestimated by CMIP6 GCMs, although the CESM2 can reproduce the stilling and reversal. Almost all state‐of‐art reanalysis products or GCMs underestimate the downward trend in NSWS,^15^, ^49^, ^50^ which may be due to the following reasons. First, the NSWS changes are sensitive to changes in surface roughness and topography.^9^ In the GCMs, each grid‐cell value represents the mean wind speed over that region. These estimates will exhibit considerably less variation in NSWS compared to that of the observed NSWS, in particular for the regions with complex terrain.^42^ Second, the observed NSWS is not assimilated in most GCMs. Assimilation of the observed wind data has been a vitally important process to improve the accuracy of GCMs in simulating the variations in NSWS.^21^ Thus, to reduce the uncertainties of GCMs in simulating the long‐term changes in NSWS, more efforts are required to improve surface process parameterization schemes and model resolution of GCMs, assimilate the observed wind data in the assimilation system, and simulate NSWS with high‐precision land cover data.^21^ Third, the performance of GCMs in simulating the trends of NSWS could be also influenced by models simulating sea surface temperature (SST) changes. The study of Zeng et al.^3^ has shown that the regions where the atmospheric simulations are forced with the observed SST capture the stilling. In contrast, regions in which the atmospheric simulations are forced without the observed SST do not capture the terrestrial stilling. Another reason behind the difference in trend magnitude between observations and models may be that the reduction in NSWS could also be influenced by anthropogenic aerosol emissions,^51^ urbanization,^40^ and LUCC.^52^ However, these factors could not be well captured by GCMs. Aerosol radiative effects alter the thermodynamic stability and convective potential of the lower atmosphere leading to reduced temperatures and increased atmospheric stability.^51^ An increase in air stability can reduce vertical mixing, which in turn decreases the vertical flux of horizontal momentum, further reducing the transfer of fast winds aloft to the surface and slowing surface winds.^53^ LUCC and urbanization can increase the surface roughness, and then increase the drag force, which reduces NSWS.^25^

## CONCLUSION

In this study, we assess the performance of the CMIP6 models in simulating long‐term changes in the global NSWS. Once the CMIP6 model with better performance in simulating past NSWS variability has been selected, future changes in NSWS have been explored together with the uncertainties and the possible causes of the projected changes. The main conclusions are shown as follows:

1. The CESM2 most accurately reproduces the observed trends of NSWS during 1978–2014. For all SSPs, the global NSWS is projected to decrease based on the CESM2. On the regional scale, the projected NSWS is characterized by decreasing trends over North America, Europe, and mid‐to‐high latitudes of Asia, and increasing trends over South Asia and Africa. For SSP585, the number of light windy days is higher, and the number of strong windy days is lower than that with SSP245.
2. There is an asymmetry change in the future NSWS between two hemispheres based on the projections of CESM2, and this spatial feature is more pronounced for SSP585 than for SSP245. Due to the discrepancies in the internal variability, the model bias, and the initial condition among models, the projected results based on a single GCM could show uncertainty. The asymmetry changes in the future NSWS between two hemispheres are also simulated by the CMIP6 models that show good performance in simulating the historical NSWS changes. Meanwhile, such hemispheric asymmetry in NSWS changes is also stimulated by MPI‐ESM LEs. Therefore, the results shown in this paper could be convincing. If only the strongest emission scenario is considered, the above‐mentioned factors could result in the reductions in NSWS over the mid‐to‐high latitudes of the NH (reduction of 1.29%, 1.89%, and 3.03% for near‐term, mid‐term, and long‐term, respectively) and the increases in NSWS over the south of 20°N (reduction of 0.03%, 0.22%, and 1.63% for near‐term, mid‐term, and long‐term, respectively).
3. The changes in SAT gradient caused by increased emissions could contribute to the asymmetry changes in future NSWS between two hemispheres. The strong warming over the high latitudes of the NH reduces the magnitude of the SAT gradient between the high and low latitudes of the NH. The magnitude of the SAT gradient decreases for all SSPs, and the SATD decline in SSP245 is weaker than that in SSP370 and SSP585. The magnitude of the SAT gradient decreases in the NH by 1.2%, 3.5%, and 4.1% and increases in the SH by 0.8%, 1.0%, and 1.5% with SSP245, SSP370, and SSP585, respectively. Therefore, we conclude that higher emission scenarios could cause opposite SAT gradient changes between the NH and SH, which induces a more significant interhemispheric asymmetry.

The results of this study provide new insights into the future changes of global terrestrial NSWS. However, we must state the limitations of our results. The difference in future changes in NSWS over two hemispheres is not homogeneous. The SAT increases with the GHG emissions, but the warming displays temporal nonsynchrony and spatial nonuniformity, and these characteristics result in spatial differences in SAT changes in the horizontal direction. Furthermore, some regional‐scale factors, such as urbanization,^9^ anthropogenic aerosol emissions,^33^ and LUCC have a considerable effect on the NSWS changes.^25^ The asymmetry changes in NSWS over two hemispheres do not mean a consistent decrease in NSWS over all regions of the NH and a consistent increase in NSWS over all regions of the SH. The effects of the regional‐scale factors on future changes in NSWS are also not isolated and quantified in the current study. Finally, GHG emissions also have a considerable effect on large‐scale ocean–atmosphere circulations (LOACs). Several previous studies have shown that the LOACs have a considerable contribution to the NSWS changes.^3^ However, the physical mechanisms of future changes in LOACs attributed to the GHGs' emission effect on the NSWS are not estimated in the current study but warrant future investigation. Nevertheless, the results presented here are a step toward improving our understanding of global terrestrial NSWS changes in the future.

## AUTHOR CONTRIBUTIONS

C.S., J.Z., and D.C. conceived the study and designed its implementation. C.S. and Z.L. performed the analysis and drafted the figures. C.S., J.Z., and Z.L. wrote the first draft of the manuscript. All authors edited, revised, and approved the final version of the manuscript.

## COMPETING INTERESTS

The authors declare no competing interests.

## Supporting information


**Table S1**: Parameters of the Coupled Model Intercomparison Project Phase 6 (CMIP6) models.
**Figure S1**: Distribution of Global Summary of Day database by the U.S. National Climate Data Center (red dots). Seven regional domains are selected for estimating the performance of CMIP6 models in this study.
**Figure S2**: Spatial patterns of the difference between the future and present‐day NSWS in CESM2‐WACCM model during (A, D, G) near‐term, (B, E, H) mid‐term, and (C, F, I) long‐term periods under (A, B, C) SSP245, (D, E, F) SSP370, and (G, H, I) SSP585. The shaded area represents the results that pass the significance *t‐*test at the 0.10 level.
**Figure S3**: Same as Figure S2, but for the MIROC6 model.
**Figure S4**: Relative to historical (1995–2014) change of zonal mean NSWS in CESM2‐WACCM model during (A, D, G) near‐term, (B, E, H) mid‐term, and (C, F, I) long‐term periods under (A, B, C) SSP245, (D, E, F) SSP370, and (G, H, I) SSP585. The horizontal axis denotes the latitudes, and the vertical axis denotes the relative historical changes in NSWS in the future.
**Figure S5**: Same as Figure S4, but for the MIROC6 model.

## Data Availability

The CMIP6 datasets are openly available at https://esgf‐node.llnl.gov/search/cmip6/. The MPI_ESM Les are openly available at https://esgf‐data.dkrz.de/projects/mpi‐ge/. The GSOD is openly available at ftp://ftp.ncdc.noaa.gov/pub/data/gsod.
