# Supplementary material for: Evaluation of global terrestrial near‐surface wind speed simulated by CMIP6 models and their future projections
Source: Ann N Y Acad Sci. 2022 Oct 14;1518(1):249–63. doi: 10.1111/nyas.14910 (PMC10092706; doi:10.1111/nyas.14910)
Supplement: Supplementary file 1 — Table S1: Parameters of the Coupled Model Intercomparison Project Phase 6 (CMIP6) models. Figure S1: Distribution of Global Summary of Day database by the U.S. National Climate Data Center (red dots). Seven regional domains are selected for estimating the performance of CMIP6 models in this study. Figure S2: Spatial patterns of the difference between the future and present‐day NSWS in CESM2‐WACCM model during (A, D, G) near‐term, (B, E, H) mid‐term, and (C, F, I) long‐term periods under (A, B, C) SSP245, (D, E, F) SSP370, and (G, H, I) SSP585. The shaded area represents the results that pass the significance t‐test at the 0.10 level. Figure S3: Same as Figure S2, but for the MIROC6 model. Figure S4: Relative to historical (1995–2014) change of zonal mean NSWS in CESM2‐WACCM model during (A, D, G) near‐term, (B, E, H) mid‐term, and (C, F, I) long‐term periods under (A, B, C) SSP245, (D, E, F) SSP370, and (G, H, I) SSP585. The horizontal axis denotes the latitudes, and the vertical axis denotes the relative historical changes in NSWS in the future. Figure S5: Same as Figure S4, but for the MIROC6 model. [file NYAS-1518-249-s001.docx]

Supplementary Information for

*Title:* **Evaluation of global terrestrial near-surface wind speed simulated by CMIP6 models and their future projections**

*Short running title:* **Global terrestrial surface winds projections**

Cheng Shen^1^, Jinlin Zha^2,3^, Zhibo Li^4^, Cesar Azorin-Molina^5^, Kaiqiang Deng^6^, Lorenzo Minola^1,5,7^, Deliang Chen^1,*^

*Surnames or family names are underlined*

^1^ Regional Climate Group, Department of Earth Sciences, University of Gothenburg, Gothenburg 40530, Sweden

^2^ Key Laboratory of Atmospheric Environment and Processes in the Boundary Layer over the Low-Latitude Plateau Region, Department of Atmospheric Science, Yunnan University, Kunming, 650091, People’s Republic of China

^3^ Key Laboratory of Regional Climate and Environment for Temperate East Asia, Institute of Atmospheric Physics, Chinese Academy of Sciences, Beijing 100029, People’s Republic of China

^4^ Laboratory for Climate and Atmosphere-Ocean Studies, Department of Atmospheric and Oceanic Sciences, School of Physics, Peking University, Beijing, 100871, People’s Republic of China

^5^ Centro de Investigaciones sobre Desertificación, Consejo Superior de Investigaciones Científicas (CIDE,CSIC-UV-*Generalitat Valenciana*), Climate, Atmosphere and Ocean Laboratory (Climatoc-Lab), Moncada, Valencia, Spain

^6^ School of Atmospheric Sciences, Sun Yat-sen University, and Southern Marine Science and Engineering Guangdong Laboratory (Zhuhai), Zhuhai, Guangdong, 519082, People’s Republic of China

^7^ Interuniversity Department of Regional and Urban Studies and Planning (DIST), Politecnico and University of Turin, Turin, Italy.

**Submitted to *the Annals of the New York Academy of Sciences***

***Special issue The Year in Climate Science Research***

*Corresponding author: Deliang Chen (deliang@gvc.gu.se)

**Table S1:** Parameters of the Coupled Model Intercomparison Project Phase 6 (CMIP6) models.

| **No.** | **Model** | **Modelling group** | **Country** | **Grid size** |
| --- | --- | --- | --- | --- |
| 1 | ACCESS-CM2 | Commonwealth Scientific and Industrial Research Organization, Australian Research Council Centre of Excellence for Climate System Science | Australia | 144×192 |
| 2 | ACCESS-ESM1-5 | Commonwealth Scientific and Industrial Research Organization | Australia | 145×192 |
| 3 | CanESM5 | Canadian Centre for Climate Modelling and Analysis | Canada | 64×128 |
| 4 | BCC-CSM2-MR | Beijing Climate Center | China | 160×320 |
| 5 | CAMS-CSM1-0 | Chinese Academy of Meteorological Science | China | 160×320 |
| 6 | CESM2 | National Center for Atmospheric Research | USA | 192×288 |
| 7 | CESM2-WACCM | National Center for Atmospheric Research | USA | 192×288 |
| 8 | EC-Earth3-Veg | EC-Earth-Consortium | EC | 256×512 |
| 9 | FGOALS-f3-L | Institute of Atmospheric Physics, Chinese Academy of Sciences | China | 180×288 |
| 10 | FGOALS-g3 | Institute of Atmospheric Physics, Chinese Academy of Sciences | China | 80×180 |
| 11 | GFDL-CM4 | National Oceanic and Atmospheric Administration, Geophysical Fluid Dynamics Laboratory | USA | 180×288 |
| 12 | GFDL-ESM4 | National Oceanic and Atmospheric Administration, Geophysical Fluid Dynamics Laboratory | USA | 180×288 |
| 13 | INM-CM4-8 | Institute for Numerical Mathematics, Russian Academy of Science | Russia | 120×80 |
| 14 | INM-CM5-0 | Institute for Numerical Mathematics, Russian Academy of Science | Russia | 120×80 |
| 15 | IPSL-CM6A-LR | Institute for Pierre Simon Laplace | France | 143×144 |
| 16 | KACE-1-0-G | National Institute of Meteorological Sciences, Korea Meteorological Administration, Climate Research | Republic of Korea | 144×192 |
| 17 | MIROC6 | Japan Agency for Marine-Earth Science and Technology, Atmosphere and Ocean Research Institute, National Institute for Environmental Studies, PIKEN Center for Computational Science | Japan | 128×256 |
| 18 | MPI-ESM1-2-HR | Max Planck Institute for Meteorology | Germany | 192×384 |
| 19 | MPI-ESM1-2-LR | Max Planck Institute for Meteorology | Germany | 96×192 |
| 20 | MRI-ESM2-0 | Meteorological Research Institute | Japan | 160×320 |
| 21 | NorESM2-LM | Center for International Climate and Environmental Research, Norwegian Meteorological Institute, Nansen Environmental and Remote Sensing Center, Norwegian Institute for Air Research, University of Bergen, University of Oslo | Norway | 96×144 |
| 22 | NorESM2-MM | Center for International Climate and Environmental Research, Norwegian Meteorological Institute, Nansen Environmental and Remote Sensing Center, Norwegian Institute for Air Research, University of Bergen, University of Oslo | Norway | 192×288 |

**
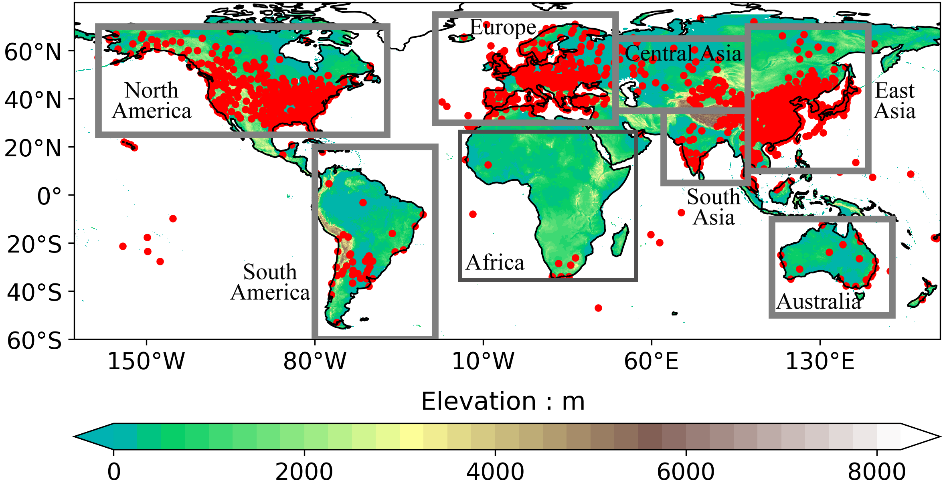
**

**Figure S1:** Distribution of Global Summary of Day database by the U.S. National Climate Data Center (red dots). Also shown are the seven regional domains selected for estimating of performance for CMIP6 models in this study. North America: 25°N-72°N, 170°W-50°W. South America: 60°S-20°N, 80°W-30°W. Europe: 30°N-75°N, 30°W-45°E. Central Asia: 30°N-65°N, 45°E-100°E. South Asia: 5°N-35N, 65°E-100°E. East Asia: 10°N-79°N, 100°E-150°E. Australia: 50°S-10°S, 110°E-160°E. Africa: 40°S-20N, 20°W-55°E.


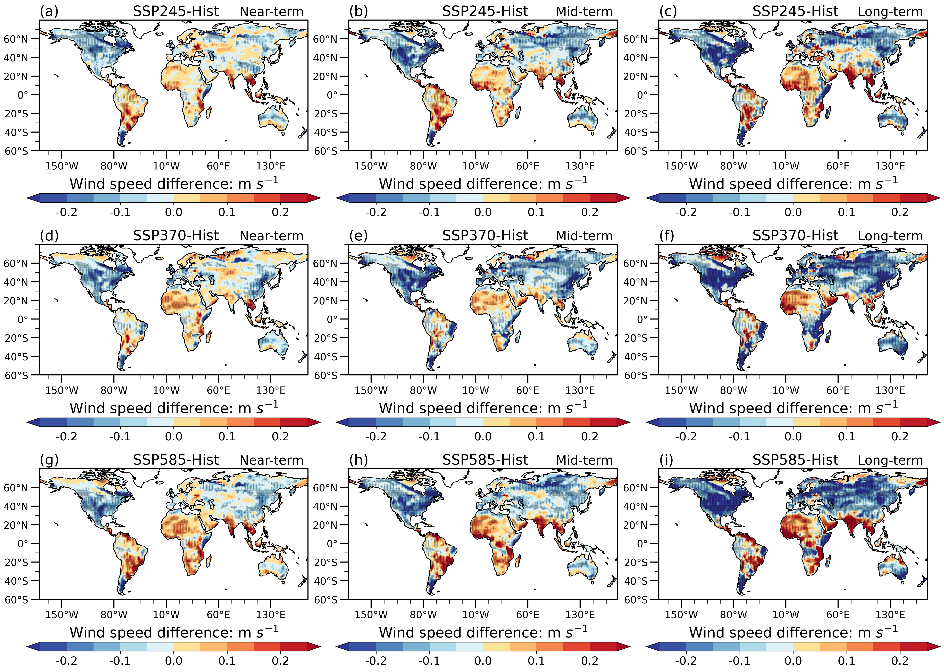


**Figure S2:** Spatial patterns of the difference between the future and present-day NSWS in CESM2-WACCM model during (a, d, g) near-term, (b, e, h) mid-term, and (c, f, i) long-term periods under (a, b, c) SSP245, (d, e, f) SSP370, and (g, h, i) SSP585. The shaded area represents the results that pass the significance *t-test* at the 0.10 level.


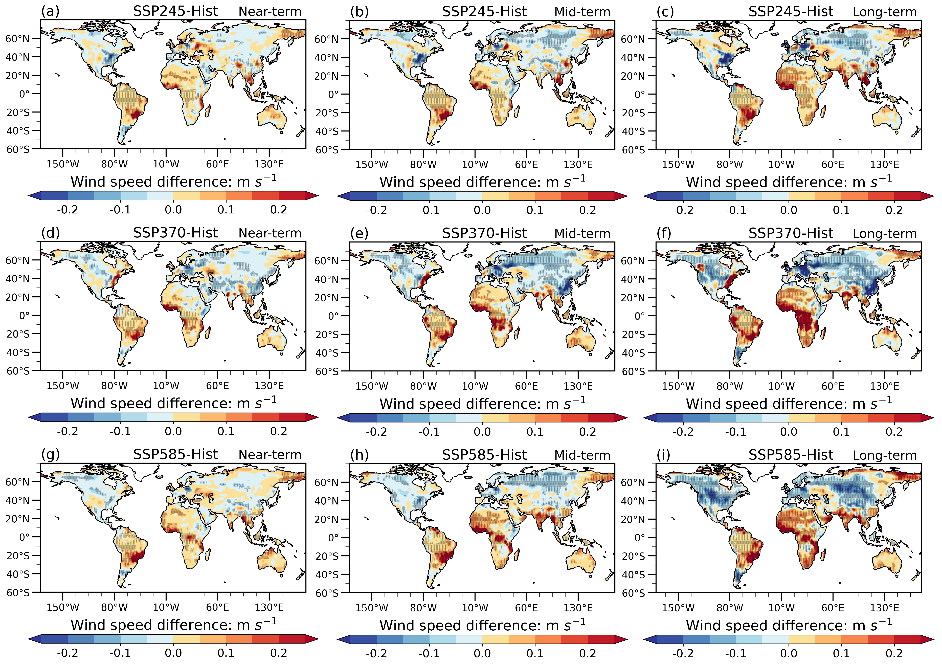


**Figure S3:** Same as Figure S2, but for the MIROC6 model.


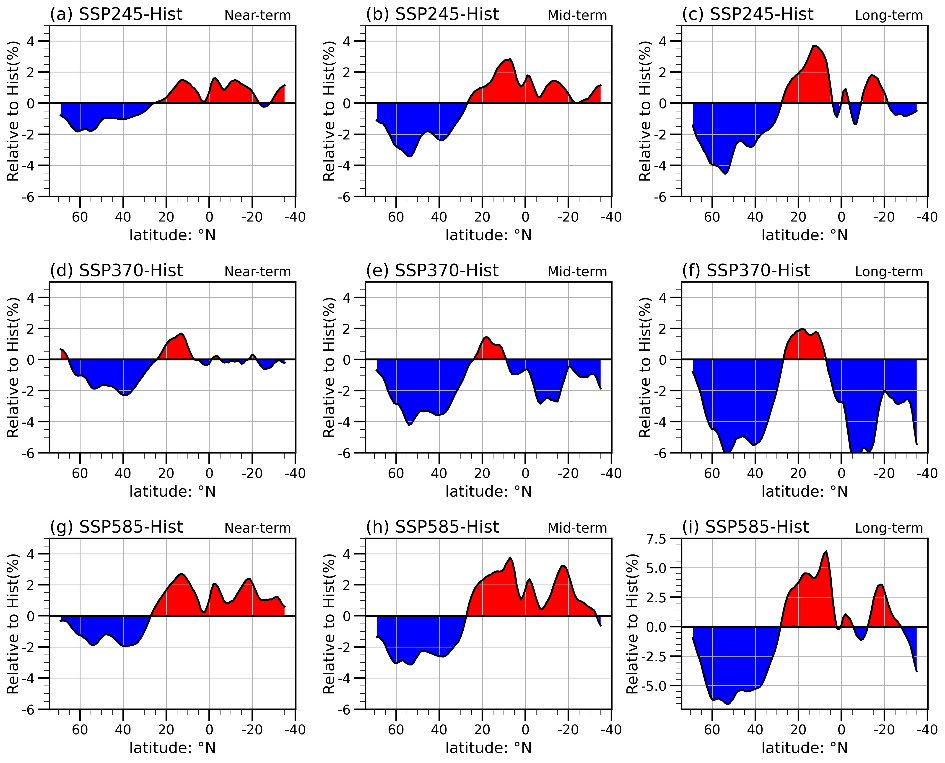


**Figure S4:** Relative to historical (1995-2014) change of zonal mean NSWS in CESM2-WACCM model during (a, d, g) near-term, (b, e, h) mid-term, and (c, f, i) long-term periods under (a, b, c) SSP245, (d, e, f) SSP370, and (g, h, i) SSP585. The horizontal axis denotes the latitudes, and the vertical axis denotes the relative historical changes in NSWS in the future.


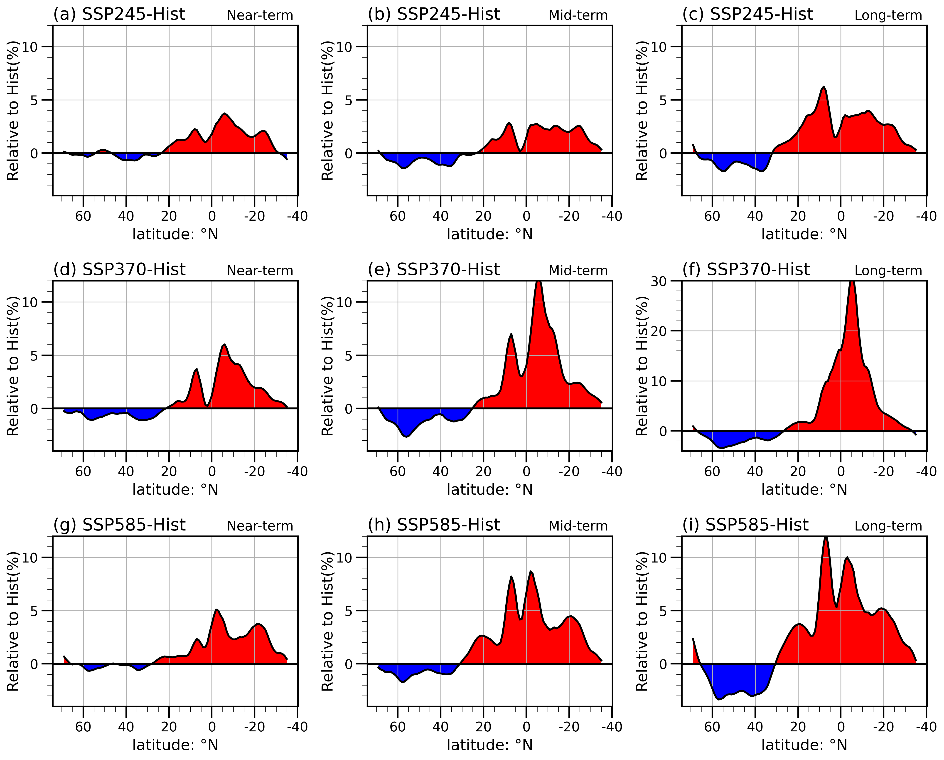


**Figure S5:** Same as Figure S4, but for the MIROC6 model.
